# Antifungal prophylaxis in high-risk patients with hematologic malignancy: a comparison of practice and guidelines in Türkiye

**DOI:** 10.55730/1300-0144.5942

**Published:** 2024-11-29

**Authors:** Özlem GÜZEL TUNÇCAN, Seçkin ÇAĞIRGAN, Hakan ÖZDOĞU, Sinem CİVRİZ BOZDAĞ, Filiz VURAL, Mustafa PEHLİVAN, Hüseyin Saffet BEKÖZ, Yasemin ESEN, Tuba KOÇ, Petek GİLİK, Esin ŞENOL

**Affiliations:** 1 Department of Infectious Diseases, Faculty of Medicine, Gazi University, Ankara, Turkiye; 2 Division of Hematology, Department of Internal Medicine, Medical Park Hospital, İzmir, Turkiye; 3 Division of Hematology, Department of Internal Medicine, Faculty of Medicine, Başkent University, Adana, Turkiye; 4 Division of Hematology, Department of Internal Medicine, Faculty of Medicine, Ankara University, Ankara, Turkiye; 5 Division of Hematology, Department of Internal Medicine, Faculty of Medicine, Ege University, İzmir, Turkiye; 6 Division of Hematology, Department of Internal Medicine, Faculty of Medicine, Gaziantep University, Gaziantep, Turkiye; 7 Division of Hematology, Department of Internal Medicine, Medipol Mega University Hospital, İstanbul, Turkiye; 8 MSD Türkiye, İstanbul, Turkiye

**Keywords:** Invasive fungal infections, graft-versus-host-disease, hematologic malignancy, antifungal prophylaxis, antifungal agents

## Abstract

**Background/aim:**

Primary antifungal prophylaxis (AFP) is as considered the gold standard therapy for patients with hematologic malignancies based on the available guidelines. The aim behind this study was to investigate the level of guideline knowledge and the translation of guideline recommendations into real-life settings among physicians in Türkiye.

**Materials and methods:**

In this prospective, observational study, the physicians’ knowledge of AFP guideline recommendations for patients with acute myeloid leukemia (AML) or myelodysplastic syndrome (MDS) undergoing first remission-induction (Group I) or graft-versus-host-disease (GvHD) after allogeneic stem cell transplant (Group II) was compared with clinical practice via an 11-item multiple-choice questionnaire evaluating the patients in whom AFP was initiated, the timing, the AFP duration, and the drugs used.

**Results:**

The mean patient age was 48.3 years and 79% had AML/MDS. AFP was initiated in 75.3% of Group I patients on the first day of induction chemotherapy before baseline and in 26.2% of Group II patients at baseline. For 98.8% of the Group I patients, the physician’s plan was to continue AFP until recovery from neutropenia and complete remission (CR), whereas it was to be used during immunosuppression in Group II. The median (IQR) duration of AFP was 0.8 (0.5–1.9) and 5.5 (2.4–8.1) months for Groups I and II, respectively. Breakthrough invasive fungal infections (bIFIs) were observed in 35.8% and 14.3% of Groups I and II, respectively.

The mean survey scores were 8.5 ± 2.3 for the AML/MDS patient group and 9.9 ± 3.2 for the GvHD patient group. Most of the surveyed sites adhered to the guidelines from the European Conference on Infections in Leukemia (80.6%), followed by the fever and neutropenia guidelines of the Infectious Diseases Society of America (51.6%). Posaconazole was the drug of choice in both groups (96.8% in Group I vs. 71% in Groups II).

**Conclusion:**

The data indicate an inconsistency between real-life clinical practice and the guideline recommendations for primary AFP and bIFI management in high-risk patients with hematologic malignancies in Türkiye.

## 1. Introduction

Invasive fungal infections (IFIs) are opportunistic infections, leading to significant morbidity and mortality rates among immunocompromised patients [1]. Depending on the presence of risk factors such as age, underlying malignancy, chemotherapy regimen, and systemic antifungal prophylaxis (AFP), the incidence of IFI varies from 2% to 49%, with a mortality rate of 60% [2]. After the introduction of routine computerized tomography examinations, the development of more effective and safer antifungal agents, and the availability of novel diagnostic techniques, such as galactomannan enzyme immunoassay, the management of IFIs in patients with hematologic malignancies has improved [2]. Furthermore, the new triazoles, such as voriconazole and posaconazole, with a wider spectrum of activity, higher efficacy, and improved safety, have recently improved the role of AFP among patients with hematologic malignancies [3]. Despite the effective reduction in overall IFIs by the administration of AFP, nearly 30% of patients still need subsequent antifungal treatment due to breakthrough IFIs (bIFIs) or insufficient/ineffective AFP [4–7]. Therefore, AFP is currently regarded as the gold standard in hematologic malignancies that have a high risk of infection, such as acute myeloid leukemia (AML), myelodysplastic syndrome (MDS), allogeneic hematopoietic stem cell transplantation (HSCT), and graft-versus-host disease (GvHD) [3,8].

The incidence of IFIs is particularly high in AML [9]. Most AML patients usually experience invasive aspergillosis after induction chemotherapy since this is their first time having profound immunosuppression [10]. The development of an IFI during the first induction generally causes major changes in the subsequent therapeutic strategy for AML. High-risk MDS and AML are treated similarly and are considered to have the same risk profile [11]. Therefore, IFIs not only affect the mortality rate but may delay treatment plans, prolong hospitalization and increase the overall disease-related expenses.

Initiating AFP is a challenging issue for the treating physicians due to additional costs, possible adverse reactions, and the development of resistance to antifungals [11]. Therefore, experts and scientific associations have published consensus reports and guidelines based on the results of prospective studies reporting that risk stratification, benefit/risk calculation, and individualization of AFP should be well-assessed in patients with hematologic malignancies [3,11–18]. Moreover, the guidelines have addressed a vast range of challenging points in AFP, such as optimal drug choice, time to start AFP, route of administration, efficacy and safety profiles, the optimal duration of AFP, follow-up procedures, and the assessment of response to therapy [9,12–14,19–21].

Despite the development of new agents, accumulated evidence in AFP, and improved healthcare technology, there are still problems in the implementation of AFP guidelines in daily clinical practice in Türkiye. Since there is insufficient data on the rate and duration of AFP in patients with hematologic malignancies, the underlying causes cannot be clearly defined. This study aimed to compare the AFP practice in patients with AML, MDS, and GvHD being monitored in tertiary healthcare centers in Türkiye versus the corresponding recommendations of the Infectious Diseases Society of America Fever and Neutropenia Guideline 2010 update (IDSA 2010) and the European Conference on Infections in Leukemia Guideline 5 (ECIL-5 2013) [14,15]. Furthermore, the AFP patterns (timing, treatment duration, and agents) used at the study centers, their efficacy of prophylaxis for survival and prevention of bIFI, and the prescribers’ self-reported knowledge of AFP guidelines for patients with AML, MDS, and GvHD versus their clinical practice were investigated.

## 2. Materials and methods

### 2.1. Study design

This was a noninterventional, multicenter, prospective, observational study on high-risk patients with hematologic malignancies. The study was performed in line with the Declaration of Helsinki and good clinical practice.

The recruitment period was 4 months. No diagnostic, prophylactic, or therapeutic suggestions were provided to the treating physicians during their daily clinical practice. The patients were treated according to the local prescribing information and routine medical practice in terms of AFP and the types of assessments performed.

A steering committee was constituted for this study and consisted of three experts in hematology. The committee developed a survey based on the recommendations of ECIL-5 2013 and IDSA 2010 for treating physicians.

The weighting factors defined for the choices were as follows: -1 indicated strongly against the recommendation, 0 meant weakly against the recommendation, 1 supported the recommendation, and 2 strongly supported the recommendation. Each choice was initially scored as 1 and then multiplied by the weighting factor to build up the total score of the treating physician. The maximum survey scores were 24 points regarding ECIL-5 2013 and 28 points for IDSA 2010. For each recommendation, the frequencies of physician choices and the frequencies of their application in real-life practice were compared.

This study was conducted in accordance with principles of good clinical practice and was approved by the Scientific Research Ethics Committee of the Gazi University Faculty of Medicine with protocol number TUTF-BAEK 2017/250 (decision no. 349, dated 30 April 2018).

### 2.2. Study population

The following inclusion criteria were applied: 1) The patients had to be at least 18 years of age. 2) The patients had a new diagnosis of AML or MDS and were receiving remission-induction treatment, or previously diagnosed AML or MDS patients developed GvHD after allogenic stem cell transplantation. 3) The patients received remission-induction treatment due to AML or MDS and expected to be neutropenic subjects (absolute neutrophil count <500/μL) for 7 days or more due to the remission-induction treatment. 4) The patients had to be able to take oral medications during the treatment duration, but if the patient needed to switched to the intravenous product, there was no restriction. 5) Each patient had to provide written informed consent prior to any study-specific procedures.

Due to the observational study design, no exclusion criteria were defined, except for pregnant and breastfeeding women. Enrolled patients who did not meet the inclusion criteria were removed from the study.

### 2.3. Data collection

This study was conducted across the hematology departments of seven tertiary healthcare centers across Türkiye where high-risk patients with hematologic malignancy were commonly treated.

At the patients’ monthly visits, data on demographics, medical history, physical examination findings, laboratory and radiological examinations, concomitant diseases and medications, previous and current treatments including the drugs and duration of use, AFP, and possible/probable/proven IFI/bIFI were collected from the medical files and recorded in case report forms. For AFP patients who developed bIFIs, the IFIs were classified as possible, probable, or proven according to the European Organization for Research and Treatment for Cancer Mycoses Study Group criteria [22].

The questionnaire, containing 11 multiple-choice questions, was developed to collect information on infectious diseases physicians’ and hematologists’ knowledge of AFP in high-risk patients with hematologic malignancy. The questionnaire was based on clinical practice recommendations according to ECIL-5 2013 and IDSA 2010 guidelines [14,15]. It was applied to physicians who were members of teams managing high-risk patients with hematologic malignancies at all sites.

### 2.4. Statistical analysis

IBM SPSS v24.0 was used to perform the statistical analysis. The MedDRA v22.0 medical dictionary was used for medical coding of the adverse events when needed. Descriptive statistics, i.e. mean, median, standard deviation, range, frequency, and percentage, were used to summarize the study data. To evaluate the relationship between various variables, contingency tables were drawn, and a chi-square test was applied where appropriate.

Possible differences between continuous variables were examined using a paired t-test or a Wilcoxon signed-rank test for related samples. A Student’s t-test or a Mann–Whitney U test was applied for independent samples.

## 3. Results

### 3.1. Patient demographics and baseline characteristics

A total of 136 patients were screened, of whom 123 were included in the statistical analysis. Thirteen patients did not meet inclusion criteria 2 and/or 3, so they were not included in the analysis. Approximately half of the patients completed the study as per protocol (61 of 123, 49.6%). The main reasons for discontinuation were the incidence of adverse events (18 of the 62 discontinued patients), IFI development (17 of 62), and death (13 of 62).

The patients with AML (n = 78, 63.4% of the study population) or MDS (n = 3, 2.4%) constituted the majority of the study population and comprised Group I (AML/MDS). Patients with GvHD after allogeneic HSCT (n = 42, 34.1%) comprised Group II. The demographic, clinical, and baseline characteristics of both groups are presented in Table 1. Most of the Group II patients had Grade 2 GvHD (20 of the 42 GvHD patients, 47.6%).

The majority of Group I patients were newly diagnosed, with a mean time from diagnosis of less than 1 month. This accounts for the low percentage of Group I patients that received at least one disease-related treatment before study enrolment (6.2%). The majority of the study population (77.2%) received at least one concomitant medication during the study, mainly proton pump inhibitors (32.3% of the study patients), serotonin antagonists (21%) and nucleoside/nucleotide reverse transcriptase inhibitors (19.4%).

The most common concurrent disease-related treatments were cytarabine (86.4%), idarubicin, and daunorubicin in Group I, and cyclosporine (61.9%,), methylprednisolone (52.4%,), and prednisolone (23.8%) in Group II.

### 3.2. AFP patterns

All patients received AFP at baseline, and 49.6% started it on the first day of induction treatment (Table 2). The planned duration of AFP for 98.8% of Group I patients was until recovery from neutropenia and complete remission, while for Group II it was throughout the immunosuppressive treatment period. Antimold prophylaxis was initiated in 44.7% of the study patients due to the expectation of prolonged neutropenia, and in 38.2% due to intensive treatment.

AFP drug switching was infrequent (13.6% of patients in Group I, and 2.4% in Group II) (Table 3). Up to two switches were reported in Group I patients. The most frequently administered drug in both groups was posaconazole followed by fluconazole (Table 3). The median (IQR) duration of AFP was longer in GvHD patients compared to AML/MDS patients (5.5 [2.4–8.1] vs. 0.8 [0.5–1.9] months, respectively). The main reasons given for AFP discontinuation were complete remission (CR) or IFI (possible/probable/proven) in Group I and IFI or a decreased glucocorticoid dose in Group II.

The emergence of bIFIs was mainly observed in Group I, at a rate of 35.8%, with fewer instances recorded for Group II (14.3%) (Table 4). Empirical therapy, mainly with caspofungin and liposomal amphotericin B, was administered to Group I and Group II patients with bIFIs. Of the Group I patients with bIFIs, the majority experienced either a complete response to the bIFI treatment (34.5%) or a failure of the bIFI treatment (44.8%). Of the Group II patients with bIFIs, 66.6% responded either partially or completely to treatment (Table 4).

During the study, one or two fever episodes were observed in 96.8% of AML/MDS patients and in 92.3% of GvHD patients (Table 1). In clinical practice, an individualized approach based on patient condition and disease severity was the most common approach for both groups (66.1% for Group I vs. 92.3% for Group II).

### 3.3. Physician adherence to recommendations

The mean total scores for patients overall and by group are given in Table 5. The physicians declared that they followed the recommendations of either the ECIL-5 2013 (80.6%) or the IDSA 2010 (51.6%) guidelines in planning AFP for AML/MDS and GvHD patients (Table 5). For patients with AML or MDS, almost all physicians (96.8%) preferred posaconazole and secondarily liposomal amphotericin B, and 77.4% of the physicians stated that they would initiate these drugs on the first day of induction treatment. For GvHD, initiation of antimold prophylaxis was preferred in the preengraftment phase (at 67.7% of treatment centers) and for patients receiving intensive immunosuppressive therapy (58.1%). The physicians’ preference for AFP in GvHD patients was posaconazole followed by fluconazole. It was also reported that majority of the physicians agreed with the continuation of AFP until recovery from neutropenia and CR for AML/MDS patients (74.2%) and throughout the immunosuppressive period for GvHD patients after allogeneic HSCT (80.6%). A combination of serum galactomannan measurements and computed tomography (64.5%) was the preferred diagnostic approach for follow-up of azole-based AFP. For febrile patients undergoing AFP, over 50% of the physicians preferred a personalized approach depending on the patient’s condition and severity of the infection, or they initiated empirical therapy with an antiaspergillus drug and, in case of no response, performed pulmonary radiography and computed tomography.

### 3.4. Adverse events during AFP

At least one AFP-related adverse event (AE) was observed in approximately half of the overall study population (Table 2). The most common AFP-related AEs in both groups were gastrointestinal. Nausea and diarrhea each affected 7.4% of Group I patients, and nausea and abdominal pain affected 14.3% and 9.5%, respectively, of Group II patients.

## 4. Discussion

IFIs are the leading cause of morbidity and mortality in patients with hematological malignancies worldwide [2,16]. The Transplant Associated Infections Surveillance Network, a consortium between the U.S. Centers for Disease Control and Prevention and 23 U.S. academic transplant centers, was launched in 2001 to determine the burden of IFI among transplant recipients. The consortium determined that the most common IFIs were invasive aspergillosis (43%) and invasive candidiasis (28%), among others (including mucormycosis at 8%) [23]. Among the AFP treatments, fluconazole has reduced the frequency of candidemia in patients undergoing HSCTs at the expense of an increase in the incidence of invasive mold infections, mainly aspergillosis [24]. Pagano et al. reported that mortality rates related to *Aspergillus* spp. and *Candida* spp. in newly diagnosed patients with hematologic malignancies (AML, lymphoma, multiple myeloma, chronic leukemia) in Italy decreased from 60%–70% down to 40% due to new management strategies and down to 30%–40% due to new treatment approaches [25]. Evidence-based guidelines have also improved the management of patients at high risk for hematologic malignancies, such as AML, MDS, and GvHD patients undergoing allogeneic HSCT [15].

The present multicenter, noninterventional study is the first to investigate the level of knowledge of AFP-relevant guidelines and the translation of these recommendations into real-life clinical practice for high-risk patients with hematologic malignancies, namely AML, MDS, and GvHD patients after allogeneic HSCT, in real-life settings in Türkiye. Real-life data from 81 AML/MDS patients and 42 GvHD patients were analyzed and compared with physician responses to a questionnaire titled “Physician’s survey on antifungal prophylaxis practice in high-risk patients with hematologic malignancy”, which was designed to reflect their knowledge about the ECIL-5 2013 and IDSA 2010 guidelines. By comparing their responses to the questionnaire to the data collected from real-life clinical practice on the case report forms, insights into physician attitudes and perspectives were obtained.

Despite the availability of various guidelines, there is no clear consensus on AFP management between different centers worldwide, particularly regarding the choice of AFP agents that are administered to high-risk patients [26,27].

The ECIL-5 2013 guidelines strongly recommend the administration of posaconazole, a second-generation azole, during intensive chemotherapy for AML patients if the baseline incidence rates of mold infections are high [15]. Alternatively, fluconazole, either alone or combined with liposomal amphotericin B, can also be used for AML or MDS patients undergoing remission-induction chemotherapy combined with biomarker screening and imaging. Conversely, the IDSA 2010 guidelines state that fluconazole, itraconazole, voriconazole, posaconazole, micafungin, and caspofungin are acceptable AFP alternatives for protection against *Candida* infections in allogeneic HSCT recipients or patients undergoing intensive remission-induction or salvage-induction chemotherapy for AML [14]. The 2024 guidelines from the National Comprehensive Cancer Network (NCCN)^1^ recommend posaconazole for patients with AML, MDS, and GvHD, fluconazole only for AML/MDS patients, and voriconazole for AML, MDS, and GvHD. Itraconazole is not recommended for AML, MDS or GvHD patients [19]. In ESCMID (European Society of Clinical Microbiology and Infectious Diseases), fluconazole and posaconazole are recommended for GvHD patients with allogeneic HSCT, whereas voriconazole and itraconazole are suggested in later ranks [15,28].

The survey results demonstrate that the majority of physicians took into account the ECIL-5 2013 guidelines followed by the IDSA 2010 guidelines in their real-life clinical practice. In agreement with the guidelines, posaconazole was the most frequently chosen treatment choice in the survey and the most frequently administered AFP for both Group I and II patients in clinical practice. Strikingly, although the physicians mostly declared that posaconazole was the preferred antifungal agent during AFP in both groups, the clinical administration rates were lower than the survey-reported rates, particularly for GvHD patients. However, in this study, the GvHD patients had lower bIFI rates than the AML/MDS group, probably because the GVHD patients who enrolled to the study all received AFP, and they received it for a longer period of time compared to the AML/MDS patients. The administration rates for liposomal amphotericin B (L-AMB) were equally lower in clinical practice compared to the guidelines, particularly for AML/MDS patients. Moreover, the timing of AFP initiation and duration of prophylaxis was not compliant with the information collected in the survey. Thus, in daily practice, primary AFP was initiated in three-fourths of patients with AML/MDS on the first day of induction treatment. Generally, in daily clinical practice, drug selection, AFP initiation time, and duration of IFI prophylaxis were more compliant with the guideline recommendations in patients with AML/MDS than in GvHD patients.

There was also an inconsistency in the physician responses and level of knowledge of guidelines regarding the follow-up of bIFIs. The median duration of AFP was shorter in patients with AML/MDS, potentially contributing to the reported rate of bIFIs in these patients that were mainly possible/probable IFI cases.

The score regarding the knowledge and IFI management obtained from the survey was low for GvHD patients and even lower for patients with AML/MDS. This finding indicates that the level of knowledge is not adequate to manage this special patient subset with a challenging treatment process.

The current guidelines and expert opinions describe the algorithms for diagnostic workups during bIFIs similarly [11,12,14,29,30]. Physicians are recommended to evaluate host defense, examine the site of infection including the central nervous system, and perform laboratory workups including serum galactomannan measurements and blood cultures. In the present study, the diagnostic work-up algorithms were performed in a few patients with bIFIs, and the results were inadequate to plan targeted treatment. The majority of bIFI cases were treated based only on host factors in addition to fever unresponsive to extensive antibiotics. Switching to the diagnostic algorithm and decision-making based on serum galactomannan measurement was the least preferred approach in bIFI cases with fever.

Primary AFP and the management of bIFIs had lower effectiveness than expected. This may be explained by the inadequate clinical implementation of AFP and diagnostic workup studies in patients with hematologic malignancies.

The adherence to AFP guidelines in high-risk patients with malignancies in the inpatient setting has been investigated in other studies worldwide. Despite the heterogeneity of the individual studies, one review of the literature concluded that given the prevalence of IFIs in malignant hematology inpatients, suboptimal adherence with antifungal guidelines is alarming and warrants caution [26]. This review demonstrated that the knowledge of antifungal guidelines is inadequate, with high rates of inappropriate antifungal prescription recommendations that ranged from 25% to 70% of the overall fungal prescriptions. Therefore, it concluded that a focus on physician education, antifungal stewardship, and an update of existing guidelines to meet real-world scenarios is mandatory. Adherence to antifungal guidelines in the outpatient hematology setting is even more complicated and under-investigated and requires further research. Contrary to what was reported in the above review, the physicians in the current study knew the corresponding AFP guidelines, but their real-life implementation was inadequate. This could potentially account for the low incidence of AEs reported in the current study. Although AEs may be under-reported in real-life conditions by patients or physicians, the reported AEs were not in line with the AEs that are expected following prolonged use of the antifungal agents that were used in the current study [24,26,31].

The observational design was an inherent limitation of this study. Therefore, the evidence was less strong compared to that generated from randomized controlled trials. However, this study aimed to collect information from real-life practice in order to contribute knowledge to planning health policy actions at the national level and further clinical studies in high-risk hematologic patients. Based on these results, a conclusive decision cannot be made about the effectiveness of agents for IFI prophylaxis. Another limitation is the physician survey and prospective nature of the study as these can influence the compliance of physicians to the guidelines. The researcher investigators’ decisions to administer AFP was based on routine medical practice and preceded any consideration of participant eligibility for enrolment in the study. The possibility that missing data or the collection of data on past medical history related to AFP contributed to potential information bias cannot be ruled out. Also, even though statistically significant differences between age and gender regarding hematologic malignancies were found, this analysis was not an objective of the study, so it cannot be conclusively attributed to any specific findings. This is another of the study’s limitations.

In conclusion, there is an inconsistency between real-world evidence and guideline recommendations in AFP patterns, including drug choice, time of onset, and treatment duration in high-risk patients with hematologic malignancies in Türkiye. The clinical practice patterns indicate that knowledge levels about guideline recommendations and diagnostic algorithms are higher than their implementation in clinical practice in the inpatient setting in Türkiye. Conclusively, further national studies should be designed to clarify the underlying reasons for the low rates of primary AFP and bIFI treatment in patients with AML, MDS, and GvHD in clinical practice.

## Figures and Tables

**Table 1:** Demographic, clinical, and baseline characteristics of the study population.

	All patients (n = 123)	AML or MDS patients (n = 81)	GvHD patients (n = 42)	p*
Sex, n (%)				
Male	66 (53.7)	37 (45.7)	29 (69.0)	
Female	57 (46.3)	44 (54.3)	13 (31.0)	0.01
Age, years				
Mean ± SD	48.3 ± 15.9	51.7 ± 16.4	41.9 ± 12.8	
Median (min, max)	51.0 (18.0, 89.0)	53.0 (19.0, 89.0)	45.0 (18.0, 61.0)	0.001
Age category, n (%)				
<50 years	59 (48.0)	32 (39.5)	27 (64.3)	
50–65 years	45 (36.6)	30 (37.0)	15 (35.7)	
≥65 years	19 (15.4)	19 (23.5)	0 (0.0)	0.001
Abnormal physical examination findings, n (%)	n = 114	n = 79	n = 35	
No	76 (66.7)	58 (73.4)	18 (51.4)	
Yes	38 (33.3)	21 (26.6)	17 (48.6)	0.022
Time from diagnosis, months	n = 123	n = 81	n = 42	
Mean ± SD	2.7 ± 8.3	0.2 ± 0.5	7.5 ± 12.9	
Median [IQR]	0.0 (0.0–1.0)	0.0 (0.0–0.0)	1.0 (0.0–10.0)	
Min, max	0.0, 54.0	0.0, 2.0	0.0, 54.0	<0.0001
Time from diagnosis, n (%)	n = 123	n = 81	n = 42	
<1 month	81 (65.8)	64 (79.0)	17 (40.5)	
1–6 months	30 (24.4)	17 (21.0)	13 (31.0)	
>6 months	12 (9.8)	0 (0.0)	12 (28.5)	

* The Mann–Whitney test was used to examine the possible differences between patients with AML, MDS, and GvHD.

AML = acute myeloid leukemia; MDS = myelodysplastic syndrome; GvHD = graft versus host disease; IQR = interquartile range; SD = standard deviation.

**Table 2:** Specifications for the initiation of AFP.

	All patients (n = 123)	AML or MDS patients (n = 81)	GvHD patients (n = 42)
AFP started at baseline visit, n (%)	123 (100.0)	81 (100.0)	42 (100.0)
Starting time of AFP			
First day of induction treatment	61 (49.6)	61 (75.3)	0 (0.0)
Azole started 24 h after last anthracycline dose	15 (12.2)	15 (18.5)	0 (0.0)
First day of persistent neutropenia	5 (4.1)	5 (6.2)	0 (0.0)
Seventh day of persistent neutropenia	0 (0.0)	0 (0.0)	0 (0.0)
Not applicable	42 (34.1)	0 (0.0)	42 (100.0)
Reason for AFP			
Induction treatment	75 (61.0)	75 (92.6)	0 (0.0)
Immunosuppressive therapy	43 (35.0)	2 (2.5)	41 (97.6)
Neutropenia expected to last >7 days	38 (30.9)	37 (45.7)	1 (2.4)
Lack of environmental protection (e.g., HEPA filter)	2 (1.6)	2 (2.5)	0 (0.0)
GvHD grade	23 (18.7)	0 (0.0)	23 (54.8)
Absolute neutrophil count	13 (10.6)	13 (16.0)	0 (0.0)
GvHD underlying hematologic malignancy	2 (1.6)	0 (0.0)	2 (4.8)
Planned duration of AFP			
Until recovery from neutropenia and complete remission	81 (65.9)	80 (98.8)	1 (2.4)
Until 5 days after complete remission	2 (1.6)	1 (1.2)	1 (2.4)
Minimum of 15 days	1 (50.0)	1 (100.0)	0 (0.0)
Minimum of 2 months	1 (50.0)	0 (0.0)	1 (100.0)
Throughout the immunosuppressive therapy	43 (35.0)	1 (1.2)	42 (100.0)
Antimold prophylaxis started at baseline visit, n (%)	113 (91.9)	79 (97.6)	34 (81.0)
Reason for antimold prophylaxis			
Expectation of prolonged neutropenia	55 (44.7)	54 (66.7)	1 (2.4)
Intensive treatment	47 (38.2)	37 (45.7)	10 (23.8)
Preengraftment allogenic HSCT	0 (0.0)	0 (0.0)	0 (0.0)
Postengraftment allogenic HSCT with GvHD	26 (21.1)	0 (0.0)	26 (61.9)
Not applicable	10 (8.1)	2 (2.5)	8 (19.1)

AML = acute myeloid leukemia; MDS = myelodysplastic syndrome; GvHD = graft versus host disease; AFP = antifungal prophylaxis; HSCT = hematopoietic stem cell transplantation; HEPA =high-efficiency particulate air; LAF = laminar airflow.

**Table 3:** Observed AFP practice patterns.

	All patients (n = 123)	AML or MDS patients (n = 81)	GvHD patients (n = 42)
Patients who switched AFP at least once during the study, n (%)	13 (10.6)	11 (13.6)	2 (2.4)
Drug switches per patient, n (%)	n = 13	n = 11	n = 2
1	9 (69.2)	7 (63.6)	2 (100.0)
2	4 (30.8)	4 (36.4)	0 (0.0)
≥3 or more	0 (0.0)	0 (0.0)	0 (0.0)
Reason for antifungal drug switch(es)			
Chemotherapy protocol	9 (7.3)	9 (11.0)	0 (0.0)
Side effects/diarrhea	4 (3.2)	3 (3.7)	1 (2.4)
Oral intake abnormality and ileus	1 (0.8)	1 (1.2)	0 (0.0)
Oral intake abnormality	1 (0.8)	1 (1.2)	0 (0.0)
Ineffective drug	1 (0.8)	0 (0)	1 (2.4)
Duration of AFP, months			
Mean ± SD	3.5 ± 6.3	1.6 ± 1.8	7.2 ± 9.5
Median [IQR]	1.5 (0.7–4.7)	0.8 (0.5–1.9)	5.5 (2.4–8.1)
Min, max	0.1, 60.1	0.1, 8.2	0.9, 60.1
Patients with ongoing AFP, n (%)	13 (10.6)	1 (1.2)	12 (28.6)
Patients with at least one temporary AFP discontinuation, n (%)	2 (1.6)	2 (2.5)	0 (0.0)
Reason for stopping AFP, n (%)			
Complete remission of primary disease	36 (29.3)	34 (42.0)	2 (4.8)
Possible/probable/proven IFI	32 (26.0)	25 (30.9)	7 (16.7)
Death	12 (9.8)	10 (12.3)	2 (4.8)
End of induction treatment	6 (4.9)	2 (2.5)	4 (9.5)
Lost to follow up	3 (2.4)	0 (0.0)	3 (7.1)
Glucocorticoid dose decreased	4 (3.3)	0 (0.0)	4 (9.5)
Neutropenic fever	4 (3.3)	3 (3.7)	1 (2.4)
Change of disease drug protocol	2 (1.6)	1 (1.2)	1 (2.4)
Immunosuppressive treatment ended	3 (2.4)	0 (0.0)	3 (7.1)
Patient request	3 (2.4)	0 (0.0)	3 (7.1)
AFP not prescribed	1 (0.8)	1 (1.2)	0 (0)
Decreased immunosuppression	1 (0.8)	0 (0.0)	1 (2.4)
Lack of drug in the center	1 (0.8)	1 (1.2)	0 (0.0)
Respiratory failure	1 (0.8)	1 (1.2)	0 (0.0)
Unknown / absent patient	1 (0.8)	1 (1.2)	0 (0.0)
Drugs used for AFP, n (%)			
Posaconazole	120 (97.6)	81 (100.0)	39 (92.9)
Fluconazole	15 (12.2)	9 (11.1)	6 (14.3)
Liposomal amphotericin B	6 (4.9)	4 (4.9)	2 (4.8)
Voriconazole	1 (0.8)	0 (0.0)	1 (2.4)

AML: acute myeloid leukemia; MDS: myelodysplastic syndrome; GvHD: graft versus host disease; IQR: interquartile range; SD: standard deviation.

**Table 4:** The efficacy of AFP during follow-up based on bIFI development.

	All patients (n = 123)	AML or MDS patients (n = 81)	GvHD patients (n = 42)
Patients with no IFI* during follow-up, n (%)	88 (71.5)	53 (64.2)	36 (85.7)
Patients with IFI* during follow-up, n (%)	35 (28.5)	29 (35.8)	6 (14.3)
IFI* classification according to EORTC-MSG criteria	n = 35	n = 29	n = 6
Possible	17 (48.6)	16 (55.2)	1 (16.7)
Proven	4 (11.4)	3 (10.3)	1 (16.7)
Probable	14 (40.0)	10 (34.5)	4 (66.6)
Antifungal treatment approach, n (%)	n = 35	n = 29	n = 6
Empirical (based only on host factors plus fever unresponsive to extensive antibiotic treatment)	25 (71.4)	22 (75.9)	3 (50.0)
Preemptive (IFI diagnosis suspected due to microbiological or clinical factors)	7 (20.0)	5 (17.2)	2 (33.3)
Targeted treatment (proven diagnosis according to EORTC-MSG criteria)	3 (8.6)	2 (6.9)	1 (16.7)
Drugs used for AFP, n (%)	n = 35	n = 29	n = 6
Caspofungin	20 (57.1)	19 (65.5)	1 (16.7)
Liposomal amphotericin B	12 (34.3)	8 (27.6)	4 (66.6)
Voriconazole	7 (20)	6 (20.7)	1 (16.7)
Posaconazole	3 (8.6)	2 (6.9)	1 (16.7)
Fluconazole	1 (2.9)	1 (3.4)	0 (0.0)
Micafungin	1 (2.9)	1 (3.4)	0 (0.0)
Same drug for AFP and IFI (dose increased), n (%)	3 (8.6)	2 (6.9)	1 (16.7)
Duration of antifungal (IFI-related) treatment			
<7 days	4 (11.4)	3 (10.3)	1 (16.7)
≥7 days	31 (88.6)	26 (89.7)	5 (83.3)
Response to antifungal treatment, n (%)			
Complete response†	12 (34.3)	10 (34.5)	2 (33.3)
Partial response‡	3 (8.6)	1 (3.4)	2 (33.3)
Stable disease§	5 (14.2)	4 (13.8)	1 (16.7)
Failure ||	14 (40.0)	13 (44.8)	1 (16.7)
Unknown	1 (2.9)	1 (3.4)	0 (0.0)

* Any IFI diagnosed on the third day or later of AFP was defined as a breakthrough IFI.

† A complete response required resolution of all clinically attributable signs and symptoms and complete resolution of radiographic or bronchoscopy abnormalities.

‡ A partial response required clinically meaningful improvement in all attributable clinical signs and symptoms, a significant improvement in radiographic (at least 50%) or bronchoscopy abnormalities, and persistence of radiographic sequelae regardless of the overall level of clinical or radiographic improvement.

§ A stable disease included no improvement of clinically attributable signs or symptoms and no improvement of radiographic or bronchoscopy abnormalities.

|| Failure included deterioration in clinically or radiographically attributable abnormalities that necessitated alternative antifungal therapy or resulted in death.

AML = acute myeloid leukemia; MDS = myelodysplastic syndrome; GvHD = graft versus host disease; IFI = invasive fungal infection; EORTC-MSG = European Organization for Research and Treatment for Cancer Mycoses Study Group.

**Table 5:** Results of the physician survey on AFP practice in high-risk patients with hematologic malignancy.

Guidelines followed in planning AFP in AML/MDS and GvHD patients, n (%)	n = 31
ECIL-5	25 (80.6)
IDSA	16 (51.6)
NCCN	3 (9.7)
EBMT	1 (3.2)
Immunosuppressive therapy	1 (3.2)
Survey questions, n (%)	
In your opinion, which of the following is/are an indication of AFP in AML/MDS patients?	
Induction treatment	31 (100.0)
Immunosuppressive therapy	13 (41.9)
Neutropenia expected to last for more than 7 days	17 (54.8)
Absence of environmental protection (HEPA filter, LAF, etc.)	10 (32.3)
In your opinion, which of the following is/are an indicator of the need for AFP in GvHD patients?	
Immunosuppressive therapy	22 (71.0)
Grade of GvHD	19 (61.3)
Absolute neutrophil count	13 (41.9)
The type of underlying hematologic malignancy	9 (29.0)
Which of the following is/are your first-choice antifungal agents for prophylaxis in AML/MDS patients?	
Micafungin	1 (3.2)
Posaconazole	30 (96.8)
Liposomal amphotericin B	30 (96.8)
Fluconazole	2 (6.5)
Voriconazole	3 (9.7)
In your opinion, when should AFP be started in AML/MDS patients?	
On the first day of induction treatment	24 (77.4)
If an azole antifungal is added to the treatment, 24 h after the last anthracycline dose	8 (25.8)
On the first day of persistent neutropenia	2 (6.5)
On the seventh day of persistent neutropenia	1 (3.2)
In your opinion, when or in whom should antimold antifungal prophylaxis be started in GvHD patients?	
Postengraftment phase	6 (19.4)
Preengraftment phase	21 (67.7)
When the patient is expected to have prolonged neutropenia	6 (19.4)
For patients receiving intensive immunosuppressive therapy	18 (58.1)
Which prophylactic antifungal agent would be your first choice in GvHD patients in the postengraftment phase?	
Micafungin	0 (0.0)
Posaconazole	22 (71.0)
Liposomal amphotericin B	4 (12.9)
Fluconazole	9 (29.0)
Voriconazole	2 (6.5)
In your opinion, at which grade(s) should AFP be started in GvHD patients?	
Grade 1	3 (9.7)
Grades 2, 3 and 4	27 (87.1)
Grades 3 and 4	4 (12.9)
Grade 4	0 (0.0)
In your opinion, which would be the proper length of AFP in AML/MDS patients?	
Until recovery from neutropenia and complete remission	23 (74.2)
Until 5 days after complete remission is achieved	5 (16.1)
Minimum 10 days	0 (0.0)
Minimum 15 days	2 (6.5)
Minimum 28 days	3 (9.7)
In your opinion, which would be the proper length of AFP in GvHD patients?	
Minimum 1 month	1 (3.2)
Minimum 2 months	0 (0.0)
Minimum 3 months	5 (16.1)
During the whole period of immunosuppressive therapy	25 (80.6)
Until 2 weeks after complete remission is achieved	5 (16.1)
Which is/are the diagnostic tools used during follow-up patients on azole-based AFP?	
No specific tool is required if there is no fever	8 (25.8)
Therapeutic drug monitoring for azole drugs, if available	3 (9.7)
Serum galactomannan measurement	7 (22.6)
Serum galactomannan measurement and computed tomography	20 (64.5)
Therapeutic drug monitoring for azole drugs in case of absorption issues	3 (9.7)
In your opinion, what should be done in case of fever in patients receiving AFP?	
Therapeutic drug monitoring for azole drugs in case of breakthrough suspicion	3 (9.7)
Empirical therapy with an antiaspergillus antifungal drug; in case of no response, pulmonary radiography and computed tomography	16 (51.6)
Individualized approach due to the patient’s condition and severity of the infection	18 (58.1)
Switch to the diagnostic algorithm, decision made based on serum galactomannan measurement	9 (29.0)
Survey total score	n = 31
All patients	
Mean ± SD	15.58 ± 4.26
Median [IQR]	15.00 (12.00–17.00)
Min, max	11.00, 28.00
Patient-related scores in AML and MDS	
Mean ± SD	8.45 ± 2.28
Median [IQR]	8.00 (7.00–9.00)
Min, max	6.00, 15.00
GvHD patient-related score	
Mean ± SD	9.94 ± 3.17
Median [IQR]	9.00 (8.00–11.00)
Min, Max	7.00, 18.00

AML = acute myeloid leukemia; MDS = myelodysplastic syndrome; GvHD = graft versus host disease; AFP = antifungal prophylaxis, ECIL = European Council on Infections in Leukemia; IDSA = Infectious Diseases Society of America; NCCN = National Comprehensive Cancer Network; EBMT = European Group for Blood and Marrow Transplantation.

## Footnotes

1 National Comprehensive Cancer Network, Inc. (2024). NCCN Clinical Practice Guidelines in Oncology (NCCN Guidelines®) for Prevention and Treatment of Cancer-Related Infections v.2.2024 © [online]. Website https://www.nccn.org/professionals/physician_gls/pdf/infections.pdf [Accessed 15 August 2024].
